# A phase Ib/II study of cabozantinib (XL184) with or without erlotinib in patients with non-small cell lung cancer

**DOI:** 10.1007/s00280-017-3283-z

**Published:** 2017-03-28

**Authors:** Heather A. Wakelee, Scott Gettinger, Jeffrey Engelman, Pasi A. Jänne, Howard West, Deepa S. Subramaniam, Joseph Leach, Michael Wax, Yifah Yaron, Dale R. Miles, Primo N. Lara

**Affiliations:** 1 Stanford Cancer Institute, 875 Blake Wilbur Drive, Rm 2233, Stanford, CA 94305-5826 USA; 2 Yale University Cancer Center, New Haven, CT USA; 3 Massachusetts General Hospital, Charlestown, MA USA; 4 Lowe Center for Thoracic Oncology, Dana Farber Cancer Institute, Boston, MA USA; 5 Swedish Cancer Institute, Seattle, WA USA; 6 Department of Hematology and Oncology, Georgetown University Hospital, Washington, DC USA; 7 Park Nicollet Cancer Center, Minneapolis, MN USA; 8 Summit Medical Group, Berkeley Heights, NJ USA; 9 Exelixis, South San Francisco, CA USA; 10 University of California Davis Comprehensive Cancer Center, Sacramento, CA USA

**Keywords:** Non-small cell lung cancer, Resistance, Cabozantinib, Erlotinib, Phase Ib/II, Combination therapy

## Abstract

**Purpose:**

Cabozantinib is a multi-kinase inhibitor that targets MET, AXL, and VEGFR2, and may synergize with EGFR inhibition in NSCLC. Cabozantinib was assessed alone or in combination with erlotinib in patients with progressive NSCLC and *EGFR* mutations who had previously received erlotinib.

**Methods:**

This was a phase Ib/II study (NCT00596648). The primary objectives of phase I were to assess the safety, pharmacokinetics, and pharmacodynamics and to determine maximum tolerated dose (MTD) of cabozantinib plus erlotinib in patients who failed prior erlotinib treatment. In phase II, patients with prior response or stable disease with erlotinib who progressed were randomized to single-agent cabozantinib 100 mg qd vs cabozantinib 100 mg qd and erlotinib 50 mg qd (phase I MTD), with a primary objective of estimating objective response rate (ORR).

**Results:**

Sixty-four patients were treated in phase I. Doses of 100 mg cabozantinib plus 50 mg erlotinib, or 40 mg cabozantinib plus 150 mg erlotinib were determined to be MTDs. Diarrhea was the most frequent dose-limiting toxicity and the most frequent AE (87.5% of patients). The ORR for phase I was 8.2% (90% CI 3.3–16.5). In phase II, one patient in the cabozantinib arm (*N* = 15) experienced a partial response, for an ORR of 6.7% (90% CI 0.3–27.9), with no responses for cabozantinib plus erlotinib (*N* = 13). There was no evidence that co-administration of cabozantinib markedly altered erlotinib pharmacokinetics or vice versa.

**Conclusions:**

Despite responses with cabozantinib/erlotinib in phase I, there were no responses in the combination arm of phase II in patients with acquired resistance to erlotinib. Cabozantinib did not appear to re-sensitize these patients to erlotinib.

**Electronic supplementary material:**

The online version of this article (doi:10.1007/s00280-017-3283-z) contains supplementary material, which is available to authorized users.

## Introduction

Erlotinib and other epidermal growth factor receptor (EGFR) tyrosine kinase inhibitors (TKIs) are established first-line treatments for patients with non-small cell lung cancer (NSCLC) with activating mutations in *EGFR*. Despite response rates of 60–70% with EGFR TKIs in this patient population, the median progression-free survival (PFS) with these agents remains under 1 year [1] due to the development of acquired resistance [2, 3] through secondary resistance mutations (e.g., T790M) [4–8] and emergence of bypass signaling pathways such as vascular endothelial growth factor (VEGF) and MET [2, 7–11]. Data suggest that dual inhibition of the VEGF and EGFR pathways can delay the development of resistance to EGFR TKIs in the upfront setting [12] but may not overcome acquired resistance [13], whereas targeting MET has been shown to re-sensitize tumors resistant to EGFR TKIs in preclinical models [14].

Cabozantinib is a small-molecule inhibitor of multiple tyrosine kinases including MET, AXL, VEGF receptors (VEGFRs), and RET [15]. The drug has been approved by the US Food and Drug Administration (FDA) for the treatment of progressive, metastatic medullary thyroid cancer [16, 17] and, more recently, for advanced renal cell carcinoma after prior anti-angiogenic therapy [18, 19].

Preclinical studies in NSCLC suggest a potential for cabozantinib to re-sensitize tumors to EGFR inhibitors. The addition of cabozantinib to gefitinib had no notable impact on activity in a gefitinib-sensitive NSCLC cell line but re-sensitized a gefitinib-resistant cell line [14]. Furthermore, the combination of cabozantinib and erlotinib demonstrated substantially increased anti-tumor activity over either agent administered alone in a MET-amplified NSCLC xenograft model resistant to EGFR inhibition.

Based on the hypothesis that the expression of MET and VEGF are mechanisms of acquired resistance to EGFR TKIs in NSCLC tumors [2, 9, 11, 20], the phase Ib/II study reported here tested whether cabozantinib, alone or in combination with continued EGFR inhibition, was tolerable and active in patients with advanced NSCLC who had progressed after benefiting from erlotinib therapy. The study was designed to first determine the maximum tolerated dose (MTD) of cabozantinib and erlotinib in combination and then to assess the safety and clinical activity of the MTD combination compared with single-agent cabozantinib.

## Patients and methods

This was a multicenter, phase Ib/II, open-label study of cabozantinib and erlotinib. All patients gave written informed consent for this trial according to international guidelines. The protocol was reviewed and treatment was monitored by institutional review boards at each participating institution, and the study adhered to the principles of the Guideline for Good Clinical Practice (International Conference on Harmonisation E6 Tripartite Guideline). The trial is registered at ClinicalTrials.gov (NCT00596648).

Eligible patients had histologically confirmed Stage IIIb or IV NSCLC. Patients were at least 18 years old, had adequate organ and marrow function with an absolute neutrophil count ≥1500/mm^3^, platelets ≥100,000/mm^3^, hemoglobin ≥9 g/dL, bilirubin ≤1.5 times the upper limit of normal (ULN), serum creatinine ≤1.5 mg/dL or calculated creatinine clearance ≥60 mL/min, and alanine aminotransferase and aspartate aminotransferase ≤2.5 times ULN. Patients with uncontrolled brain metastases, clinically significant hemoptysis, and hematemesis were excluded. Patients were also excluded for the presence of cavitary pulmonary lesion(s), endobronchial lesion or a lesion abutting a major blood vessel, pregnancy or lactation, serious intercurrent illness, uncontrolled hypertension [sustained blood pressure (BP) readings of >140 mmHg systolic or >90 mmHg diastolic], unhealed wounds from recent surgery, or congestive heart failure, unstable angina, or clinically significant cardiac arrhythmias within 3 months, or transient ischemic attack, stroke, or myocardial infarction within 6 months. Anti-cancer therapy, other than erlotinib (or gefitinib in phase II), was not allowed within 4 weeks prior to entry.

In addition to these eligibility criteria, patients enrolled in phase I must have failed prior treatment with erlotinib but tolerated erlotinib at a dose greater than or equal to the dose of the cohort in which they were assigned. Patients had to have an Eastern Cooperative Oncology Group (ECOG) performance status of ≤2.

For phase II, patients had to have measurable disease per Response Evaluation Criteria in Solid Tumors (RECIST) guidelines, version 1.0 [21] and an ECOG performance status of ≤1. Patients were also required to have had progressed during treatment with erlotinib after either an initial response or at least 6 months of stable disease. Patients were also required to have amylase and lipase <1.5 times the ULN. Patients were excluded for prior therapy with a VEGFR TKI, an investigational EGFR TKI, a MET inhibitor, recent history (3 months) of radiation therapy other than to bones, or a history of idiopathic pulmonary fibrosis or interstitial lung disease.

## Treatment plan and study design

In both phase I and II, erlotinib and cabozantinib were given orally (po) once daily (qd). Cabozantinib was supplied as 20- and 80-mg capsules (expressed as the freebase equivalent weight). Erlotinib 25-, 100-, and 150-mg tablets were available commercially. Screening was conducted within 28 days before the first dose of study drug. Each cycle was 4 weeks (28 days).

Safety was assessed at frequent intervals by standard clinical and laboratory tests, physical examinations, and 12-lead electrocardiograms. All adverse events (AEs) were graded according to the National Cancer Institute Common Terminology Criteria for Adverse Events (NCI-CTCAE), version 3.0. Radiographic tumor assessments were conducted at baseline (within 7 days before first dose in phase II) and every 8 weeks after the initiation of study treatment per RECIST guidelines, version 1.0 [21].

Phase I was a 3 + 3 dose escalation/de-escalation design in 2 parallel arms: Arm A maximized the dose of cabozantinib [to a maximum dose of 140 mg qd (the single-agent MTD)] [22], and Arm B maximized the dose of erlotinib [to a maximum dose of 150 mg qd (the label dose for NSCLC)] [23]. Primary objectives in phase I were to evaluate the safety and tolerability of cabozantinib and erlotinib administered in combination, to determine a MTD of the combination, and to characterize the pharmacokinetic (PK) parameters of single-agent erlotinib (run-in period) and cabozantinib in combination with erlotinib. Exploratory objectives included the assessment of objective response rate (ORR). Erlotinib was administered as a single agent on days −14 to −1 (run-in period) at the dose used during subsequent treatment with the combination. After PK evaluation of erlotinib alone on day 1, cabozantinib was administered in combination with erlotinib qd in 28-day cycles.

Dose-limiting toxicity (DLT) was defined as any of the following that occurred during the first 28 days of treatment: a treatment-emergent AE that warranted dose reduction or was of significant risk; non-hematologic significant grade 3/4 toxicity, including grade 3 diarrhea despite prophylaxis and other optimal treatment; intolerable rash; grade 4 thrombocytopenia, grade 4 neutropenia of >5 days duration or grade 3/4 neutropenia with fever and documented infection; inability to take >75% of planned study dose owing to an AE; or inability to start Cycle 2 within 28 days of planned start date because of an AE. Events clearly unrelated to either cabozantinib or erlotinib were not considered DLTs. The MTD was defined as a dose level below the maximum administered dose in which <30% of the total patients in the expanded cohort experienced a DLT.

For the initial dose cohort (Cohort 1), the dose levels for the combination were cabozantinib 60 mg qd + erlotinib 150 mg qd (Table 1). The protocol was subsequently amended to allow for the 2 parallel-dose de-escalation/dose escalation arms. In Arm A, the dose of erlotinib was reduced below the approved/label dose of 150 mg qd (initially to 100 mg qd) and the initial dose of cabozantinib was maintained at 60 mg qd and increased as tolerated in subsequent cohorts to a maximum of 140 mg qd. In Arm B, the dose of erlotinib was maintained at the approved/label dose of 150 mg qd and the dose of cabozantinib was 40 mg qd. At the investigator’s discretion, patients could continue to receive cabozantinib after the DLT evaluation period either alone or in combination with erlotinib, provided that there was no evidence of progressive disease (PD) or unacceptable study drug-related toxicity.

**Table 1:** Phase I dose-level cohorts

Cohort	Initial dose (mg)^a^
1	60 cabozantinib, 150 erlotinib
2A	60 cabozantinib, 100 erlotinib
2B	40 cabozantinib, 150 erlotinib
3A	100 cabozantinib, 100 erlotinib
4A	100 cabozantinib, 50 erlotinib

^a^All cabozantinib doses are expressed as the freebase equivalent weight

Phase II was a Simon optimal 2-stage design in which patients were randomized in a 1:1 fashion to receive single-agent cabozantinib at 100 mg qd or the combination of cabozantinib 100 mg qd plus erlotinib 50 mg qd based on the phase I Arm A MTD. The phase I MTD of Arm B was not further evaluated in phase II. Initial enrollment in each arm (Stage I) was estimated at 15 patients to ensure 12 were evaluable for response. If there was ≥1 responder in Stage I, enrollment could be expanded in that arm (Stage II). In phase II, the primary objective was to estimate the ORR. Secondary objectives included the evaluation of safety and tolerability, evaluation of PFS, and characterization of PK parameters of cabozantinib and erlotinib.

Crossover was permitted during phase II at the discretion of the investigator. Patients treated in the single-agent cabozantinib arm who developed PD had the option to cross over to receive combination cabozantinib/erlotinib, whereas those receiving cabozantinib/erlotinib who experienced PD had the option to cross over to single-agent cabozantinib (at a dose greater than what they were receiving in the combination). Patients who crossed over could continue until they experienced unacceptable toxicity or treatment failure as defined by investigator assessment.

## Pharmacokinetics

Blood samples for PK assessments were taken at predetermined intervals. In phase I, PK assessments were conducted at 15 min before dose and then after dose at 30 min and 1, 2, 4, 8, and 24 h on day −1 and C2D1 (day 29); before dose and 4 h after dose on C2D15 (day 43); before dose approximately every 2 cycles (every 8 weeks) starting with C3D1 (day 57) to coincide with routine tumor assessments; and at a 30-day post-treatment visit or study withdrawal. In phase II, blood samples were collected 15 min before dose and 4 h after dose on C1D1, C1D15, C2D1, and C2D15, then every 2 cycles (8 weeks) starting with C3D1 to coincide with routine tumor assessments, ideally at the time of withdrawal from the trial.

Cabozantinib and erlotinib concentrations in plasma were determined using a validated liquid chromatography–mass spectrometry method at Exelixis, Inc. All concentrations were reported in units of ng freebase/mL. The lower limit of quantification for both analytes was 0.5 ng freebase/mL. Data assembly was performed using S-Plus® 8.0 for Windows (Enterprise Version, Tibco Software Inc., Palo Alto, CA). WinNonlin Professional 5.2 (Pharsight Corp., Mountain View, CA) was used for calculating the summary statistics and generating raw tables. Concentration–time profiles and other PK-related plots were generated using S-Plus Version 8.0 for Windows.

### Statistical considerations

The safety population consists of all patients who received study treatment. The efficacy-measurable population was defined as patients with measurable disease at baseline. The safety population was used for all analyses except ORR, which used the efficacy-measurable population. Best overall response and ORR were summarized using frequency counts and percentages, and the 90% confidence interval was computed using the exact binomial distribution. Median duration of response and the associated 90% confidence intervals (CIs) were calculated using the Kaplan–Meier method.

For patients who had a crossover in phase II, both efficacy and safety data are summarized up to the time of crossover. For all other patients, study data are summarized up to 30 days after the last dose of study drug.

Statistical analyses of safety, PK, and pharmacodynamic data were performed with WinNonlin Enterprise, version 5.0.1 (Pharsight Corp, Mountain View, CA), GraphPad Prism (version 4.02) software, SoftMaxPro GxP (version 5), and/or SAS (version 9.1).

## Results

A total of 65 patients were enrolled in phase I, and 64 patients received combination treatment with cabozantinib/erlotinib and were included in the analysis. One patient received erlotinib during the 14-day run-in period but withdrew owing to PD before receiving cabozantinib. Baseline characteristics are summarized in Table 2. In phase I, the median patient age was 59.8 years (range 30–88 years), and 44 patients (68.8%) were female. The majority of patients (43/64; 67.2%) were white; 17 (26.6%) were Asian. At baseline, all patients except 2 had an ECOG performance status of 0 or 1. The majority of patients (82.8%) had adenocarcinoma. All patients had metastatic disease at study entry for an average of >2 years from initial diagnosis, including 14% with brain metastases. The majority of patients in phase I had at least 3 anti-cancer regimens before enrolling in the study, and 63 patients (98.4%) had previously been treated with erlotinib.

**Table 2:** Demographic and baseline characteristics: phase I and II (safety populations)

	Phase I **(** *N* = 64)	Phase II	
		Cabozantinib (*N* = 15)	Cabozantinib/erlotinib (*N* = 13)
Age (years)			
Median (range)	59.8 (30–88)	54.7 (36–74)	64.8 (44–78)
Age category, *n* (%)			
18–<25 years	0	0	0
25–<45 years	6 (9.4)	2 (13.3)	1 (7.7)
45–<65 years	35 (54.7)	10 (66.7)	6 (46.2)
≥65 years	23 (35.9)	3 (20.0)	6 (46.2)
Sex, *n* (%)			
Male	20 (31.3)	3 (20.0)	6 (46.2)
Female	44 (68.8)	12 (80.0)	7 (53.8)
ECOG performance status, *n* (%)			
0	26 (40.6)	5 (33.3)	2 (15.4)
1	36 (56.3)	10 (66.7)	11 (84.6)
2	1 (1.6)	0	0
Missing	1 (1.6)	0	0
Race, *n* (%)			
Asian	17 (26.6)	4 (26.7)	3 (23.1)
Black or African American	2 (3.1)	1 (6.7)	1 (7.7)
White	43 (67.2)	10 (66.7)	8 (61.5)
Not reported	1 (1.6)	0	1 (7.7)
Other	1 (1.6)	0	0
Histology, *n* (%)			
Adenocarcinoma	53 (82.8)	14 (93.3)	11 (84.6)
Squamous cell carcinoma	3 (4.7)	1 (6.7)	2 (15.4)
Large cell carcinoma	1 (1.6)	0	0
Other	7 (10.9)	0	0

*ECOG* Eastern Cooperative Oncology Group

In phase II, 28 patients received treatment with single-agent cabozantinib (*n* = 15) or cabozantinib/erlotinib (*n* = 13). The median age was 54.7 years (range 36–74 years) in the cabozantinib arm and 64.8 years (range 44–78 years) in the combination arm. Twelve patients (80.0%) in the cabozantinib arm and 7 patients (53.8%) in the combination arm were female. The majority of patients were white [10/15 (66.7%) cabozantinib arm and 8/13 (61.5%) combination arm]; 4 (26.7%) in the cabozantinib arm and 3 (23.1%) in the combination arm were Asian. The majority of patients (93.3% receiving cabozantinib and 84.6% in the combination arm) had adenocarcinoma. All patients had metastatic disease at study entry, including 25% with brain metastases. Similar to phase I, the mean years since the initial diagnosis of metastasis was >2 years, with the majority having received at least 3 prior anti-cancer regimens. An activating *EGFR* mutation was detected in 10 of the patients in the cabozantinib arm and 7 in the combination arm; *EGFR* mutation status was unknown for the remaining 11 patients.

## Phase I MTD

DLTs from phase I of the trial are shown in Table 3. Fifteen patients experienced a DLT. Diarrhea was the most frequently observed DLT across all cohorts (10 patients experienced a DLT of diarrhea ranging from grade 2 to 3). Two of 3 patients enrolled in Cohort 1 (60-mg cabozantinib/150-mg erlotinib) experienced a DLT of diarrhea (1 with grade 2 and 1 with grade 3). The patient with grade 3 diarrhea also experienced grade 3 aspartate aminotransferase elevation and grade 3 palmar-plantar erythrodysesthesia syndrome (PPES). In Cohort 2A (60-mg cabozantinib/100-mg erlotinib), 5 of 16 patients experienced DLTs: 4 patients with grade 3 diarrhea and 1 with grade 2 diarrhea. In Cohort 3A (100-mg cabozantinib/100-mg erlotinib), 5 of 15 patients experienced a DLT: grade 3 diarrhea and grade 3 fatigue (*n* = 1), grade 3 fatigue (*n* = 1), grade 3 lipase elevation (*n* = 1), grade 3 hypertension (*n* = 1), and grade 3 hypokalemia (*n* = 1). The Cohort 3A dose exceeded the defined MTD. A total of 14 patients were enrolled in Cohort 4A (100-mg cabozantinib/50-mg erlotinib), with no patients experiencing a DLT. Based on the frequencies of DLTs in Arm A, the Cohort 4A dose level was declared the MTD of Arm A.

**Table 3:** Cabozantinib phase I DLTs

Cohort	Dose (mg)		*N*	Patients with DLTs	DLTs^a^
	Cabozantinib	Erlotinib			
1	60	150	3	2	Diarrhea, grade 2 Diarrhea, grade 3 Increase AST, grade 3 PPES, grade 3
2A	60	100	16	5	Diarrhea, grade 3 (*n* = 4) Diarrhea, grade 2
2B	40	150	17	3	Diarrhea, grade 3 (*n* = 2) Mucositis, grade 3
3A	100	100	15	5	Hypokalemia, grade 3 Hypertension, grade 3 Diarrhea, grade 3 Fatigue, grade 3 (*n* = 2) Lipase, grade 3 Epigastric pain, grade 3
4A	100	50	14	0	N/A

*AST* aspartate aminotransferase, *DLT* dose-limiting toxicity, *PPES* palmar-plantar erythrodysesthesia syndrome

^a^One event for each DLT unless otherwise specified

In Arm B, 17 patients were enrolled in Cohort 2B (40-mg cabozantinib/150-mg erlotinib), 3 of whom experienced DLTs: grade 3 diarrhea (*n* = 2) and grade 3 stomatitis (*n* = 1). The Cohort 2B dose level was determined to be the MTD of Arm B.

## Safety and tolerability

### Phase I

Treatment-emergent AEs (any grade) reported by at least 15% of patients in phase I are summarized in supplementary Table S1. The most common (>35%) AEs of any grade were diarrhea, decreased appetite, fatigue, nausea, rash, and weight decrease. Twenty patients (31.3%) had a ≥30-mmHg increase in systolic BP from baseline, and 17 patients (26.6%) had a ≥20-mmHg diastolic BP increase from baseline (supplementary Table S2). Grade 3/4 AEs were experienced by 87.5% of patients (Table 4). Diarrhea (45.3%), fatigue (21.9%), and hypokalemia (14.1%) were the most common grade 3/4 events. Twelve patients (18.8%) experienced an AE leading to drug discontinuation, with diarrhea and PPES being the most common (10.9 and 3.1%, respectively).

**Table 4:** Treatment-emergent grade 3 or 4 adverse events reported by ≥5% of patients: phase I (safety population)

Preferred term, *n* (%)	*N* = 64
Any treatment-emergent adverse event	56 (87.5)
Diarrhea	29 (45.3)
Fatigue	14 (21.9)
Hypokalemia	9 (14.1)
Dyspnea	7 (10.9)
Pneumonia	6 (9.4)
Decreased appetite	5 (7.8)
Hypophosphatemia	5 (7.8)
Hypertension	5 (7.8)
Pulmonary embolism	5 (7.8)
Weight decreased	5 (7.8)
Dehydration	4 (6.3)
Hyponatremia	4 (6.3)
Hypoxia	4 (6.3)
Pain	4 (6.3)
Back pain	3 (4.7)
Hypomagnesemia	3 (4.7)
Blood potassium decreased	3 (4.7)
Lipase increased	3 (4.7)
Renal failure acute	3 (4.7)
Palmar-plantar erythrodysesthesia syndrome	3 (4.7)
Thrombocytopenia	3 (4.7)

There were 13 deaths within 30 days of the last dose of study treatment. One death was attributed to respiratory arrest and another to asystole/coronary artery atherosclerosis and cardiac arrest; both were considered unrelated to study treatment. The remaining 11 deaths were attributed to PD. One patient who died of PD experienced a fatal pulmonary hemorrhage (day 26 after the last dose), which was assessed as possibly related to study treatment. Three additional grade 5 AEs reported in patients who died of PD were pneumonia, cardio-respiratory arrest, and respiratory failure, all unrelated to study treatment.

### Phase II

The most commonly reported AEs (>35%) in phase II were fatigue, diarrhea, nausea, decreased appetite, and PPES (supplementary Table S3). Differences were noted between treatment arms in the frequency of some AEs, including diarrhea (46.7% cabozantinib vs 84.6% cabozantinib/erlotinib), dehydration (13.3 vs 46.2%), vomiting (20.0 vs 38.5%), cough (53.3 vs 7.7%), and constipation (40.0 vs 15.4%). Eleven patients (73.3%) in the cabozantinib arm and 13 patients (100%) in the combination arm experienced at least 1 grade 3 or 4 AE (Table 5), with 53.3 and 84.6% experiencing a treatment-related grade 3 or 4 AE, respectively. Rates of grade 3/4 AEs differed between single-agent cabozantinib vs the combination for diarrhea (0 vs 30.8%), dehydration (0 vs 23.1%), and lymphopenia (0 vs 15.4%). Four patients (26.7%) in the cabozantinib arm and 3 (23.1%) in the combination arm had a ≥30-mmHg systolic BP increase from baseline; 8 patients (53.3%) and 4 patients (30.8%), respectively, had a ≥20-mmHg diastolic BP increase from baseline (supplementary Table S4).

**Table 5:** Treatment-emergent grade 3 or 4 adverse events in ≥2 patients: phase II (safety population)

Preferred term, *n* (%)	Cabozantinib (*N* = 15)	Cabozantinib + erlotinib (*N* = 13)
Hypertension	2 (13.3)	1 (7.7)
Hyponatremia	2 (13.3)	1 (7.7)
Fatigue	2 (13.3)	1 (7.7)
Hypokalemia	1 (6.7)	2 (15.4)
Diarrhea	0	4 (30.8)
Dehydration	0	3 (23.1)
Lymphopenia	0	2 (15.4)

The rate of dose modification (dose interruption or reduction) due to AEs was 66.7% for cabozantinib and 69.2% for cabozantinib/erlotinib and was primarily due to PPES (33.3 vs 23.1%) and diarrhea (6.7 and 38.5%). The most common reason for treatment discontinuation was PD (80.0% on cabozantinib and 46.2% on the combination). Discontinuation due to AEs occurred in 2 (13.3%) patients receiving cabozantinib (fatigue and non-fatal hemorrhagic stroke) and 5 patients (38.5%) receiving the combination (PPES, large intestine perforation, fatigue/diarrhea/weight loss, transient ischemic attack, and intracranial hemorrhage). Three patients in the cabozantinib arm died within 30 days of receiving their last dose of study drug, all from PD. There were 2 deaths from PD and clinical deterioration within 30 days of last receiving the combination study dose that were not considered to be related to study treatment. The intracranial hemorrhage leading to discontinuation in the combination arm was considered treatment-related and was fatal.

## Efficacy

### Phase I

The efficacy-measurable population included 61 patients across the dose cohorts. Five patients experienced a partial response (PR) for an ORR of 8.2% [90% confidence interval (CI) 3.3, 16.5%], with no apparent trend by dose level. The median PFS was 3.7 months (95% CI 3.2, 5.5 months).

The overall median duration of treatment for cabozantinib was 3.6 months (range 0.1–36.0 months), or approximately 4 cycles of study treatment. The majority of patients (57.8%) were treated until disease progression per RECIST guidelines, version 1.0. Cumulatively, there were 45 patients (70.3%) who were on study treatment for ≥8 weeks (2 cycles) and 31 patients (48.4%) on study treatment for ≥16 weeks (4 cycles). One patient with prolonged stable disease and 1 patient with prolonged objective response (each with a PFS duration of >1000 days and on study treatment >35 cycles) were eventually transitioned onto a cabozantinib maintenance protocol and continued on therapy for an additional 18+ months.

### Phase II

One of 15 patients in the single-agent cabozantinib arm achieved a confirmed PR for an ORR of 6.7% (90% CI 0.3, 27.9%), whereas there were no responses in the cabozantinib/erlotinib arm. The median PFS was 1.9 months (95% CI 1.6, 7.1 months) for the cabozantinib arm and 3.9 months (95% CI 1.5, 7.3 months) for the combination arm.

The overall median duration of treatment for cabozantinib (not including treatment after crossover) was 2.1 months (range 0.2–16.6 months) or approximately 2 cycles of study treatment in both treatment arms. Cumulatively, there were 18 patients (64.3%) who were on study treatment for ≥8 weeks (2 cycles), 12 patients (42.9%) on study treatment for ≥12 weeks (3 cycles), and 9 patients (32.1%) on study treatment for ≥16 weeks (4 cycles).

Eight patients crossed over from cabozantinib to cabozantinib/erlotinib after experiencing PD. The patient who achieved a PR with single-agent cabozantinib progressed and crossed over to receive cabozantinib/erlotinib (PFS duration of 16.6 months prior to crossover) and subsequently transitioned to the cabozantinib maintenance protocol continuing treatment for an additional 18+ months. Despite the PR in the single-agent cabozantinib arm, expansion of the arm did not proceed owing to logistical issues and prioritization of resources and not because of unexpected safety concerns or lack of efficacy.

## Pharmacokinetics

During phase Ib of the study, available PK results were analyzed for each dosing cohort at the time of the cohort review committee meetings to assess for any apparent drug–drug interaction between cabozantinib and erlotinib. Co-administration of cabozantinib (in a dose range of 40–100 mg) and erlotinib (in a dose range of 50–150 mg) had no apparent effect on plasma area under the curve of each agent compared with that agent administered alone.

Formal PK analysis was conducted in phase II of the study. In phase II, with all patients in the single-agent arm receiving cabozantinib 100 mg po qd, the mean ± standard deviation (SD) pre-dose plasma concentration of cabozantinib was 996 ± 513 ng/mL and 846 ± 335 ng/mL on day 15 (C1D15) and day 29 (C2D1), respectively. Dose normalization of these means (i.e., dividing by 100 mg) resulted in values that were generally consistent with dose-normalized results seen for other cabozantinib studies [22]. Steady state was reached by about day 15. Approximately fourfold plasma accumulation of cabozantinib was observed in this study based on the day 29:day 1 concentration at 4 h post-dose ratio, which was generally consistent with the results reported in other studies. Plasma concentrations of cabozantinib were maintained over an extended dosing period (through study day 60) for both the single-agent cabozantinib and cabozantinib/erlotinib arms during phase II (data not shown). No marked difference in plasma cabozantinib concentration was seen between the cabozantinib vs cabozantinib/erlotinib arms; there was no evidence to suggest that co-administration of erlotinib markedly altered cabozantinib PK.

Interpatient variability (coefficient of variance, CV%) in erlotinib pre-dose concentrations (79.8 and 59.8% on day 15 and day 29, respectively) was consistent with the published values [24, 25]. Furthermore, the mean dose-normalized erlotinib pre-dose concentration on day 15 (i.e., 355 ng/mL for 50-mg erlotinib = 7.1 ng/mL/mg) was generally consistent with the published results for steady-state erlotinib minimum concentration (*C*_min_), in which a mean value of approximately 7.5 ng/mL/mg was observed across the 50–200 mg erlotinib dose groups on day 24 [24, 25]. Taken together, there was no evidence to suggest that co-administration of cabozantinib markedly altered erlotinib PK.

## Discussion

Multiple strategies to overcome resistance to EGFR TKIs have been explored over the past decade. The multi-TKI cabozantinib, with potent MET/AXL/VEGFR2 blockade, seemed an ideal compound alone or in combination with erlotinib to overcome secondary erlotinib resistance. As discovered in this phase Ib/II trial, however, this strategy resulted in increased frequency of overlapping toxicities, most notably, diarrhea. Nevertheless, phase Ib established 2 combination MTDs to bring forward into future trials. Dose escalation was explored in 2 parallel arms, each maximizing doses of cabozantinib (Arm A) and erlotinib (Arm B). Dose escalation was limited predominantly by diarrhea. Eventually, the MTD for the combination of cabozantinib/erlotinib in Arm A was determined to be 100-mg cabozantinib/50-mg erlotinib (maximizing the cabozantinib dose) and the MTD in Arm B was determined to be 40-mg cabozantinib/150-mg erlotinib (maximizing the erlotinib dose). These MTDs were below the maximum planned doses, but were based on the toxicities observed in this trial. Other toxicities were as expected for a combination of EGFR and VEGFR inhibition, including PPES and hypertension. PK analysis revealed no evidence that co-administration of the agents markedly altered the PK of either drug, despite both being CYP3A4 substrates.

Phase II evaluated single-agent cabozantinib at 100 mg and the combination MTD of Arm A from phase I (100-mg cabozantinib/50-mg erlotinib). Although there was a PR in the single-agent cabozantinib arm of phase II, expansion of that arm into the second stage of enrollment did not occur for logistical reasons, preventing further assessment of single-agent activity. Toxicity was as expected in phase II. The most frequent grade 3/4 AEs were hypertension, hyponatremia, and fatigue for the cabozantinib arm and diarrhea and dehydration for the cabozantinib/erlotinib arm. The most frequent AE leading to study drug modification in the cabozantinib arm was PPES, whereas diarrhea, dehydration, and PPES were the most frequent AEs leading to dose modification in the combination arm.

One treatment-related death occurred in the phase II cabozantinib/erlotinib arm from an intracranial hemorrhage. Two other patients experienced non-fatal central nervous system events, 1 with a transient ischemic attack (combination arm) and 1 with a hemorrhagic stroke (cabozantinib). In phase Ib, 1 patient who died of PD had a potential treatment-related toxicity of pulmonary hemorrhage nearly a month after study drug discontinuation. Severe hemorrhage is a rare but potentially fatal complication that has been reported with cabozantinib in other clinical studies; thrombotic events have also been reported [26]. Thus, caution is warranted with this combination.

The ORR for phase I was 8.2% (5 of 61 patients) with no apparent dose–response trend among the combination cohorts, but there were no PRs in the 13 patients randomized to the combination arm in phase II. There was 1 responder out of 15 (6.7%) in the phase II single-agent cabozantinib arm. Almost all patients enrolled in this study were previously treated with erlotinib; however, only patients enrolled in phase II were required to have previously experienced a response or prolonged stable disease and subsequently progressed during erlotinib treatment (acquired resistance). The addition of cabozantinib to erlotinib in the phase II combination arm did not restore sensitivity to EGFR TKI therapy, although this arm was relatively small.

The limited efficacy of the cabozantinib/erlotinib combination in phase II is perhaps not surprising given the information that has emerged since the initiation of this trial implicating secondary mutations in *EGFR* (e.g., T790M) as a more common resistance mechanism than *MET* amplification (the rationale for this study) [7]. Other trials focused on MET inhibition to prevent or delay EGFR TKI resistance have also had limited success [27]. Thus, a study limitation is the lack of routine testing for *EGFR*-activating mutations, which was not standard at the time this trial was conducted (first patient enrolled February 12, 2008). We did not verify patient *EGFR*-activating mutation status, as patients were selected based on clinical criteria of response to EGFR TKI (a reasonable surrogate) or at least 6 months of stable disease on erlotinib before progression, which may have allowed a significant number of patients without true *EGFR*-activating mutations onto the trial.

As the patients enrolled in the current study were likely heterogeneous with respect to *EGFR* mutation status and mechanisms of resistance to prior EGFR TKI treatment, it is difficult to draw firm conclusions about the clinical activity of cabozantinib in *EGFR* mutant NSCLC. The responses seen with the combination in phase I and the response seen with single-agent cabozantinib in phase II supported additional investigations of cabozantinib in NSCLC. A randomized discontinuation trial of cabozantinib in unselected patients with NSCLC reported a single-agent response rate of 10% [28]. The California Cancer Consortium completed a phase II study of 37 patients with NSCLC and an *EGFR* mutation who had progressed on prior EGFR TKI therapy using the combination dose of 40 mg qd for cabozantinib and 150 mg qd for erlotinib [29]. The reported toxicities in this phase II trial were similar to those reported in the current study. The response rate was 5.4% and the disease control rate (defined as PR or SD ≥8 weeks) was 67.6%. Furthermore, the role of VEGF inhibition to delay erlotinib resistance in patients with *EGFR* mutation NSCLC recently received renewed interest with publication of a positive phase II trial looking at the addition of the VEGF antibody bevacizumab to single-agent erlotinib as a first-line strategy [12]. Taken together, further exploration of cabozantinib in combination with erlotinib in patients with *EGFR* mutations is warranted, possibly selecting patients based on MET expression.

Cabozantinib, with and without erlotinib, also holds promise in other subsets of NSCLC. Activity with single-agent cabozantinib has been reported in a small series of patients with NSCLC with translocations in *ROS1* [30] or *RET* mutations [31]. Specific trials in these patient populations are ongoing or planned. A phase II trial (E1512) focused on patients with *EGFR* wildtype disease randomized patients to either erlotinib alone (150 mg po qd), cabozantinib alone (60 mg po qd), or in combination (150 mg erlotinib/40 mg cabozantinib), with crossover to the combination for those with PD on either single agent. The study showed a significant improvement in PFS and OS with the cabozantinib-containing arms compared with single-agent erlotinib; follow-up studies are currently in development [32]. Correlative analyses of outcomes with *MET, KRAS*, and other known driver mutations are ongoing. These additional studies will provide further insight into a potential role for cabozantinib in the treatment of NSCLC.

## Electronic supplementary material

Below is the link to the electronic supplementary material.

Supplementary material 1 (DOCX 44 KB)
