# Supplementary material for: A phase Ib/II study of cabozantinib (XL184) with or without erlotinib in patients with non-small cell lung cancer
Source: Cancer Chemother Pharmacol. 2017 Mar 28;79(5):923–32. doi: 10.1007/s00280-017-3283-z (PMC5403837; doi:10.1007/s00280-017-3283-z)
Supplement: Supplementary file 1 — Supplementary material 1 (DOCX 44 KB) [file 280_2017_3283_MOESM1_ESM.docx]

A Phase Ib/II Study of Cabozantinib (XL184) with or without Erlotinib in Patients with Non‑Small Cell Lung Cancer

Heather A. Wakelee^1^, Scott Gettinger^2^, Jeffrey Engelman^3^, Pasi A. Jänne^4^, Howard West^5^, Deepa S. Subramaniam^6^, Joseph Leach^7^, Michael Wax^8^, Yifah Yaron^9^, Dale R. Miles^9^, Primo N. Lara, Jr.^10^

^1^Stanford University Cancer Center, Stanford, CA, USA; ^2^Yale University Cancer Center, New Haven, CT, USA; ^3^Massachusetts General Hospital, Charlestown, MA, USA; ^4^Lowe Center for Thoracic Oncology, Dana Farber Cancer Institute, Boston, MA, USA; ^5^Swedish Cancer Institute, Seattle, WA, USA; ^6^Department of Hematology & Oncology, Georgetown University Hospital, Washington, DC, USA; ^7^Park Nicollet Cancer Center, Minneapolis, MN, USA; ^8^Summit Medical Group, Berkeley Heights, NJ, USA; ^9^Exelixis, South San Francisco, CA, USA; ^10^University of California Davis Comprehensive Cancer Center, Sacramento, CA, USA

Corresponding Author: Heather A. Wakelee, MD

Stanford University/Stanford Cancer Center

875 Blake Wilbur Drive, Rm 2233

Stanford, CA 94305-5826

Phone (650) 723-9094; Fax (650) 724-3697; hwakelee@stanford.edu

Acknowledgements: Supported by Exelixis. Also supported in part by CTSA award number UL1 RR025744 (STANFORD CTRU) from the National Center for Research Resources, National Institutes of Health

These data were presented in part at the American Society for Clinical Oncology (ASCO) annual meeting in June 2010, Chicago, IL.

Short Title: Phase Ib/II trial of cabozantinib/erlotinib

Journal Submission: Cancer Chemotherapy and Pharmacology

Supplement

Table S1 Treatment-emergent adverse events reported by ≥15% of patients in Phase I (safety population)

| **Preferred term, n (%)** | **Cohort 01 (N=3)** | **Cohort 2A (N=15)** | **Cohort 2B (N=17)** | **Cohort 3A (N=15)** | **Cohort 4A (N=14)** | **Total (N=64)** |
| --- | --- | --- | --- | --- | --- | --- |
| At least 1 event | 3 (100.0) | 15 (100.0) | 15 (88.2) | 15 (100.0) | 14 (100.0) | 62 (96.9) |
| Diarrhea | 3 (100.0) | 14 (93.3) | 13 (76.5) | 12 (80.0) | 12 (85.7) | 54 (84.4) |
| Decreased appetite | 0 | 7 (46.7) | 11 (64.7) | 10 (66.7) | 10 (71.4) | 38 (59.4) |
| Fatigue | 2 (66.7) | 8 (53.3) | 7 (41.2) | 10 (66.7) | 10 (71.4) | 37 (57.8) |
| Nausea | 2 (66.7) | 7 (46.7) | 7 (41.2) | 11 (73.3) | 6 (42.9) | 33 (51.6) |
| Rash | 0 | 9 (60.0) | 7 (41.2) | 6 (40.0) | 3 (21.4) | 25 (39.1) |
| Weight decreased | 0 | 5 (33.3) | 7 (41.2) | 5 (33.3) | 6 (42.9) | 23 (35.9) |
| Vomiting | 1 (33.3) | 4 (26.7) | 8 (47.1) | 5 (33.3) | 4 (28.6) | 22 (34.4) |
| Dysgeusia | 0 | 3 (20.0) | 2 (11.8) | 8 (53.5) | 5 (35.7) | 18 (28.1) |
| Dizziness | 0 | 5 (33.3) | 2 (11.8) | 3 (20.0) | 7 (50.0) | 17 (26.6) |
| Dehydration | 0 | 5 (33.3) | 3 (17.6) | 5 (33.3) | 3 (21.4) | 16 (25.0) |
| Hypomagnesemia | 1 (33.3) | 4 (26.7) | 2 (11.8) | 5 (33.3) | 4 (28.6) | 16 (25.0) |
| Hypokalemia | 0 | 4 (26.7) | 1 (5.9) | 6 (40.0) | 4 (28.6) | 15 (23.4) |
| Dry skin | 2 (66.7) | 4 (26.7) | 1 (5.9) | 0 | 4 (28.6) | 11 (17.2) |
| Dyspepsia | 1 (33.3) | 2 (13.3) | 1 (5.9) | 3 (20.0) | 4 (28.6) | 11 (17.2) |
| Blood lactate dehydrogenase increased | 1 (33.3) | 2 (13.3) | 3 (17.6) | 2 (13.3) | 2 (14.3) | 10 (15.6) |
| Myalgia | 0 | 1 (6.7) | 3 (17.6) | 4 (26.7) | 2 (14.3) | 10 (15.6) |
| Stomatitis | 0 | 1 (6.7) | 3 (17.6) | 3 (20.0) | 3 (21.4) | 10 (15.6) |

**Table S2** Substantial changes from baseline in BP in Phase I

| **n (%)** | **Cohort 01   (N=3)** | **Cohort 2A   (N=15)** | **Cohort 2B  (N=17)** | **Cohort 3A  (N=15)** | **Cohort 4A  (N=14)** | **Total  (N=64)** |
| --- | --- | --- | --- | --- | --- | --- |
| ≥30-mm Hg SBP increase | 0 | 7 (46.7) | 3 (17.6) | 7 (46.7) | 3 (21.4) | 20 (31.3) |
| ≥30-mm Hg SBP decrease | 1 (33.3) | 0 | 3 (17.6) | 2 (13.3) | 3 (21.4) | 9 (14.1) |
| ≥20-mm Hg DBP increase | 0 | 6 (40.0) | 3 (17.6) | 3 (20.0) | 5 (35.7) | 17 (26.6) |
| ≥20-mm Hg DBP decrease | 1 (33.3) | 0 | 2 (11.8) | 2 (13.3) | 2 (14.3) | 7 (10.9) |

*BP* blood pressure; *DBP* diastolic BP, *SBP* systolic BP

Table S3 Treatment-emergent adverse events reported by ≥2 patients in Phase II (safety population)

| **Preferred term, n (%)** | **Cabozantinib (N=15)** | **Cabozantinib + erlotinib (N=13)** |
| --- | --- | --- |
| At least 1 event | 15 (100.0) | 13 (100.0) |
| Fatigue | 13 (86.7) | 10 (76.9) |
| Diarrhea | 7 (46.7) | 11 (84.6) |
| Nausea | 8 (53.3) | 7 (53.8) |
| Decreased appetite | 7 (46.7) | 5 (38.5) |
| Palmar-plantar erythrodysesthesia syndrome | 6 (40.0) | 4 (30.8) |
| Cough | 8 (53.3) | 1 (7.7) |
| Constipation | 6 (40.0) | 2 (15.4) |
| Dehydration | 2 (13.3) | 6 (46.2) |
| Headache | 4 (26.7) | 4 (30.8) |
| Vomiting | 3 (20.0) | 5 (38.5) |
| Hypertension | 3 (20.0) | 4 (30.8) |
| Weight decreased | 3 (20.0) | 4 (30.8) |
| Dysgeusia | 3 (20.0) | 3 (23.1) |
| Dyspnea | 4 (26.7) | 2 (15.4) |
| Edema peripheral | 3 (20.0) | 3 (23.1) |
| Rash | 2 (13.3) | 4 (30.8) |
| Dry skin | 4 (26.7) | 1 (7.7) |
| Dysphonia | 2 (13.3) | 3 (23.1) |
| Hypomagnesemia | 1 (6.7) | 4 (30.8) |
| Abdominal distension | 3 (20.0) | 1 (7.7) |
| Chest pain | 3 (20.0) | 1 (7.7) |
| Dizziness | 3 (20.0) | 1 (7.7) |
| Hypokalemia | 1 (6.7) | 3 (23.1) |
| Hyponatremia | 2 (13.3) | 2 (15.4) |
| Lipase increased | 3 (20.0) | 1 (7.7) |
| Mucosal inflammation | 1 (6.7) | 3 (23.1) |
| Oropharyngeal pain | 2 (13.3) | 2 (15.4) |
| Pain in extremity | 2 (13.3) | 2 (15.4) |
| Alopecia | 0 | 3 (23.1) |
| Anxiety | 3 (20.0) | 0 |
| Arthralgia | 2 (13.3) | 1 (7.7) |
| Aspartate aminotransferase increased | 2 (13.3) | 1 (7.7) |
| Back pain | 2 (13.3) | 1 (7.7) |
| Blood alkaline phosphatase increased | 2 (13.3) | 1 (7.7) |
| Breath sounds abnormal | 0 | 3 (23.1) |
| Confusional state | 1 (6.7) | 2 (15.4) |
| Insomnia | 3 (20.0) | 0 |
| Musculoskeletal pain | 2 (13.3) | 1 (7.7) |
| Neutropenia | 1 (6.7) | 2 (15.4) |
| Upper respiratory tract infection | 2 (13.3) | 1 (7.7) |
| Urinary tract infection | 2 (13.3) | 1 (7.7) |
| Abdominal discomfort | 2 (13.3) | 0 |
| Abdominal pain | 2 (13.3) | 0 |
| Depression | 2 (13.3) | 0 |
| Dry mouth | 1 (6.7) | 1 (7.7) |
| Dysarthria | 1 (6.7) | 1 (7.7) |
| Dysphagia | 0 | 2 (15.4) |
| Exfoliative rash | 1 (6.7) | 1 (7.7) |
| Gait disturbance | 1 (6.7) | 1 (7.7) |
| Hypophosphatemia | 0 | 2 (15.4) |
| Lymphopenia | 0 | 2 (15.4) |
| Mental status changes | 1 (6.7) | 1 (7.7) |
| Myalgia | 1 (6.7) | 1 (7.7) |
| Neck pain | 2 (13.3) | 0 |
| Neuropathy peripheral | 1 (6.7) | 1 (7.7) |
| Oral pain | 1 (6.7) | 1 (7.7) |
| Pleural effusion | 1 (6.7) | 1 (7.7) |
| Pneumonia | 1 (6.7) | 1 (7.7) |
| Productive cough | 1 (6.7) | 1 (7.7) |
| Pulmonary embolism | 1 (6.7) | 1 (7.7) |
| Rhinitis allergic | 1 (6.7) | 1 (7.7) |
| Stomatitis | 2 (13.3) | 0 |
| Transaminases increased | 1 (6.7) | 1 (7.7) |
| Vision blurred | 1 (6.7) | 1 (7.7) |
| White blood cell count decreased | 1 (6.7) | 1 (7.7) |

**Table S4** Substantial changes from baseline in BP in Phase II

| **n (%)** | **Cabozantinib alone (N=15)** | **Cabozantinib + erlotinib  (N=13)** |
| --- | --- | --- |
| ≥30-mm Hg SBP increase | 4 (26.7) | 3 (23.1) |
| ≥30-mm Hg SBP decrease | 1 (6.7) | 0 |
| ≥20-mm Hg DBP increase | 8 (53.3) | 4 (30.8) |
| ≥20-mm Hg DBP decrease | 0 | 2 (15.4) |

*BP* blood pressure; *DBP* diastolic BP, *SBP* systolic BP
